# Characterization of Terpene Synthases Reveals the Diversity of Terpenoids in *Andrographis paniculata*

**DOI:** 10.3390/molecules30102208

**Published:** 2025-05-18

**Authors:** Junhao Tang, Ying Ma, Yujun Zhao, Xiaohui Ma, Jian Wang

**Affiliations:** 1 College of Traditional Chinese Medicine, Yunnan University of Chinese Medicine, Kunming 650500, China; tangjunhao@163.com; 2 State Key Laboratory for Quality Ensurance and Sustainable Use of Dao-di Herbs, National Resource Center for Chinese Materia Medica, China Academy of Chinese Medical Sciences, Beijing 100700, China; xiaoma1110@126.com (Y.M.); zhaoyj@nrc.ac.cn (Y.Z.); 3 Key Laboratory of Yunnan Provincial Department of Education on Substance Benchmark Research of Ethnic Medicines, Yunnan University of Chinese Medicine, Kunming 650500, China

**Keywords:** *Andrographis paniculata*, diterpene synthase, sesquiterpene synthases, andrographolide, mutation

## Abstract

Terpenoids have significant biological activity and good clinical efficacy and are important for defence and physiological regulation in plants. Andrographolide and similar labdane-related diterpenoids have been isolated and characterized as the main medicinal constituents of drugs from *Andrographis paniculata*. To better study the diversity of terpenoids of *A. paniculata*, a total of 39 *ApTPSs* were screened, and 27 full-length genes encoding ApTPSs were obtained. The results showed that ApTPS4 could convert GGPP to *ent*-CPP and that ApTPS5 could convert *ent*-CPP to kaurene. This study first identified six sesquiterpene synthases with biological activity and also indicated the presence of sesquiterpenes with multiple skeletons in *A. paniculata*. The increase in the number of *ent*-copalyl diphosphate synthases and the loss of biological function by most sesquiterpene synthases and monoterpene synthases may explain why diterpenoids are the main specific metabolites in *A. paniculata* compared with the metabolites produced by AtTPSs found in the *Arabidopsis thaliana* genome. As revealed by site-directed mutagenesis, 533Val of ApTPS16 is an important site for maintaining the single main product capability, and 534Tyr of ApTPS17 may also be more important. The ApTPS17 Y534V mutation caused it to lose its main biological function. This study characterized a novel *ent*-copalyl diphosphate synthase and six sesquiterpene synthases. This provided evidence for the existence of other terpenoids and revealed the diversity of chemical components, providing a reference for future pharmacological research for *A. paniculata*.

## 1. Introduction

*Andrographis paniculata* (Burm. f.) Nees, known as ‘king of bitters’, belongs to the Acanthaceae family. It is widely used as a traditional medicine in southern and southeastern Asian countries and has a long history for the treatment of sore throat, flu, upper respiratory tract infections and other disorders [1,2]. It is utilized in the treatment of colds accompanied by fever, swollen throat, oral ulcers, etc., and as well as abscesses and ulcers and snake and insect bites. The extract exhibits pharmacological effects including anti-inflammatory, antimicrobial, hepatoprotective, and anticancer properties.

Terpenoids are one of the key active ingredients in the main medicinal activity of *A. paniculata*. Therefore, compared with flavonoids, xanthoquinones and other compounds [3], terpenoids in *A. paniculata* are the most widely and thoroughly studied. Among them, sulfonated derivatives of diterpenoid Andrographolide have been developed into traditional Chinese medicine injections, and Andrographolide and its structural analogues have also been proven to have significant biological activity, including anticancer [4], antiviral [5] and antimicrobial [6] activities. Its biosynthetic pathway is catalyzed by the diterpene synthase ApCPS2 and a series of P450s such as CYP72A399 and CYP72F1 from the CYP72 subfamily [7].

Many plant-derived terpenoids, such as diterpenoid taxol [8] and tanshinone [9], sesquiterpenoid artemisinin [10] and (-)-*β*-elemene [11] and monoterpenoid paeoniflorin [12] have significant biological activity and good clinical efficacy. In the study of the biosynthetic pathways of these terpenoids, researchers have reported and developed a series of structurally similar terpenoid active derivatives and catalytic tools such as terpenoid synthases and P450s for efficient biosynthesis. Therefore, studying terpenoids and their diversity in medicinal plants is of great significance for the exploration of new drug sources and novel biological catalytic elements. However, except for labdane-related diterpenes, there are few reports on other types of terpenoids in *A. paniculata*, which limits the research and development of its terpenoid diversity.

The diversity formation of terpenoid compounds is mediated by terpene synthase (TPSs), and the catalytic function of TPSs is closely related to their domains and motifs. TPSs are classified into two distinct categories (class I TPSs and class II TPSs) based on the presence of the conserved motifs ‘DDxxD’ or ‘DxDD’. They can be further subdivided into seven subfamilies, TPS-a, -b, -c, -d, -e, -f and -g [13], according to their amino acid sequence similarities. Class I TPSs contain ‘DDxxD’ and ‘DTE/NSE’ motifs; members include those in the TPS-a, -b, d, -e/f and -g subfamilies, which utilize a trinuclear metal to trigger the ionization of the isoprenoid substrate (e.g., GPP, FPP or GGPP) prenyl diphosphate group and generate a carbocation intermediate to initiate catalysis [14], and activity of the TPS-e/f subfamily members specific for class I diTPSs that are process-catalyzed by GGPP or CPP, resulting in the production of diterpenoid carbon skeletons [15,16]. The TPS-c subfamily contains a ‘DxDD’ motif specific for class II TPSs, which facilitates the generation of different bicyclic diphosphate intermediates through the protonation-dependent cyclization of GGPP [15,16,17].

As the functions of TPSs are continually being identified, the functions of each subfamily of TPSs are becoming increasingly diverse. Five diTPSs of *A. paniculata* have been reported in previous studies, showing that three class II diTPSs can convert GGPP to *ent*-CPP or *normal*-CPP, and two class I diTPSs can convert *ent*- or *normal*-copalyl diphosphate into different diterpenoid skeletons [18,19,20]. To date, except for diterpene synthase, key genes involved in the formation of other types of terpenoids such as monoterpenes and sesquiterpenes in *A. paniculata* have not been identified. Therefore, this study aims to explore these genes through phylogenetic analysis, conserved domain and intron-exon structure analysis, and study of the diversity of terpene compounds through the functional identification of terpene synthases in *A. paniculata*.

## 2. Results

The whole-genome database search of *A. paniculata* revealed multiple genes with functional annotations. Thirty-nine genes were selected (Table S1), all of which were annotated as terpene synthases. Thirty-five of the thirty-nine *ApTPS* genes had ORFs longer than 1500 bp and were selected for further study. We designed specific primers for *ApTPS1* through *ApTPS35* (Table S1). The *ApTPS* genes exhibited genomic sequence lengths ranging from 2015 to 14,471 base pairs, with corresponding protein lengths varying between 493 and 1141 amino acids (Table S1).

### 2.1. Phylogenetic Analysis of ApTPSs

Terpene synthases can be classified into three distinct classes according to their protein folds and different substrate catalytic mechanisms [21,22,23], which include ionization and induced carbocation formation (class I), protonation and induced carbocation formation (class II), or the use both of these mechanisms (bifunctional enzymes) [24]. All class I terpene synthases contain two conserved motifs, aspartate-rich regions (DDxxD) and (N,D)Dxx(S,T)xxxE, which utilize a trinuclear metal to trigger the ionization of the isoprenoid substrate prenyl diphosphate group and generate a carbocation intermediate to initiate catalysis [14]. Class II terpene synthases initiate the cyclization of GGPP through protonation, leading to the formation of a tertiary carbocation. This reactive intermediate subsequently undergoes a series of carbon–carbon bond-forming reactions, ultimately producing a bicyclic product. The general catalytic acid responsible for protonation is a central aspartate residue located within the conserved DxDD sequence motif [14,24].

**Figure 1:**
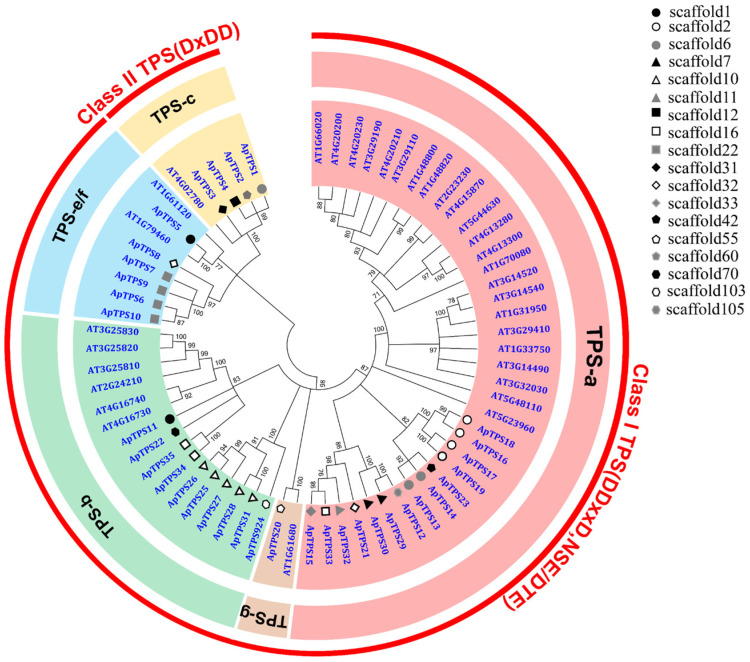
Phylogenetic tree of *A. paniculate* TPSs and those from other species. The tree was generated using the neighbour-joining method (bootstrap replications = 1000) and was condensed by a cut-off value of 70%. (These terpene synthase protein sequences of *A. thaliana* were extracted from TAIR (http://www.arabidopsis.org, accessed on 14 April 2025, Table S1).

**Figure 2:**
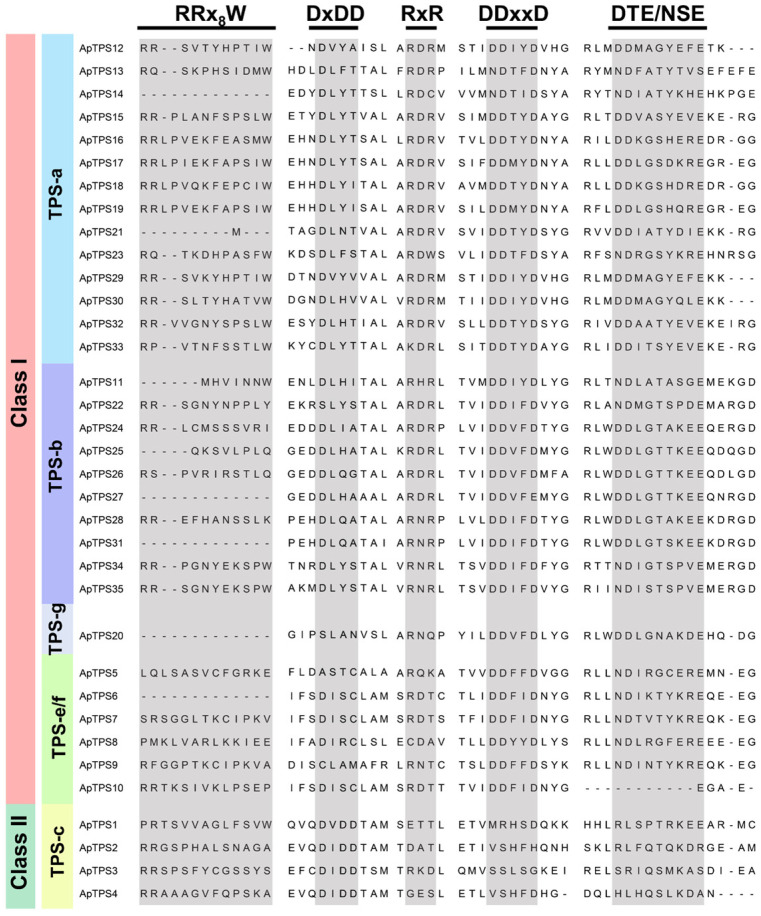
The DxDD, DDxxD, RxR, RRx8W and NSE/DTE conserved motifs of ApTPSs.

By performing a phylogenetic analysis of ApTPSs, we found that ApTPS1, ApTPS2, ApTPS3 and ApTPS4 clustered with the characterized protein (AtCPS; AT4G02780) within the TPS-c subfamily belonging to class II diTPS (Figure 1), and contained the conserved motifs DxDD (Figure 2). The biological function of the two ApTPSs (ApTPS1 and ApTPS2) has been characterized; they convert GGPP to *ent*-CPP and were named ApCPS1 and ApCPS2 [19,25], and ApTPS3 is similar to ApCPS3 [19,20,25] according to BLAST results, but the ApTPS4 enzymes of four class II TPS members have not been reported. And ApTPS5 to ApTPS35 were characterized as class I TPSs (Figure 1), which contained conserved DDxxD (or NDxxD) and clustered with the class I TPSs of *Arabidopsis thaliana*. Six of thirty-one class I TPSs clustered with the characterized AtKS genes within the TPS-e/f subfamily, which were characterized as encoding *ent*-kaurene synthase, and the members of this subfamily gene were also named class I diTPSs. Fourteen or ten of thirty-one class I TPSs clustered with the members of the TPS-a or TPS-b subfamilies of *A. thaliana*.

Seven ApTPSs groups (e.g., group 1: TPS6, 7, 9, 10; group 2: TPS16, 17, 18, 19; group 3: TPS13, 14; group 4: TPS29, 30; group 5: TPS20, 24; group 6: TPS25, 26, 27, 28, 31; group 7: TPS34, 35) were found through the analysis results of scaffold classification, which also contained the similar intron–exon structure and the conserved motifs (Figure S1), and the same groups’ ApTPSs (except group 5) were clustered in the same branch. These results reveal that these ApTPSs have a relatively close evolutionary relationship and similar catalytic activities.

### 2.2. Molecular Cloning of the Full-Length cDNA Encoding ApTPSs

The full-length coding sequences (CDSs) of 27/35 ApTPSs were amplified by PCR, with cDNA used as a template. The ApTPS information and functional annotation data are shown in Table S1. Gene-specific primers were designed to isolate ApTPSs based on the information of the genome sequence data (Table S1). The sequences of ApTPS11, ApTPS14 and ApTPS19 contained stop codons at their 5′ end, and ApTPS8, ApTPS9, ApTPS10, ApTPS21 and ATPS22 did not contain the full-length coding sequences. We performed studies on 27 ApTPSs with full-length sequences to determine their biological function.

### 2.3. Functional Identification of Recombinant Class II diTPS (TPS-c Subfamily)

**Figure 3:**
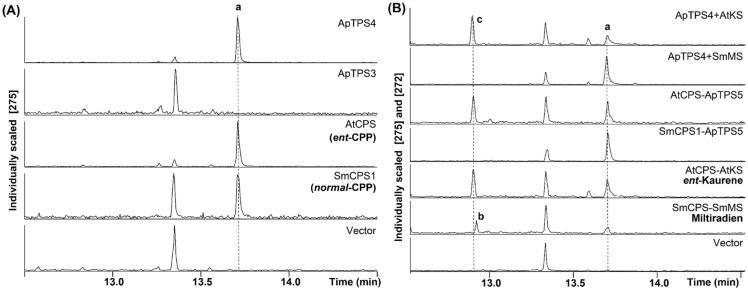
GC-MS analysis of the products from the extractions of class II diterpene synthase (in BY-T20) fermentation products. (**A**) GC-MS analysis of the dephosphorylated reaction products from the extractions of ApCPS3v2 or ApCPS4 (in BY-T20) fermentation products compared with those from CPP produced by AtCPS or SmCPS1, which is detected in this work as the hydrolysate of *ent*-/*nor*-CPP; (**B**) GC-MS analysis of ApCPS4 coupled with either AtKS or SmMS (in BY-T20) coupled with either AtCPS or SmCPS1, compared with *ent*-kaurene or miltiradiene produced by the complementary enzymatic pairs AtCPS and AtKS or SmCPS1 and SmMS, respectively; **a** (*ent*-/*normal*-)copalol (from dephosphorylated (*ent*-/*nor*-)CPP); **b** miltiradiene; **c** *ent*-kaurene.

Putative class II diTPSs were expressed in BY-T20 to characterize their biological function. The hydrolysate was detected by GC-MS (Thermo Fisher Scientific, Waltham, MA, USA) analysis. The production of CPP was detected for ApTPS4 by comparison with the known activity of AtCPS (AT4G02780) [26] and SmCPS1 (AWM30276.1) (Figure 3A), and no new products were detected for ApTPS3. ApTPS4 was coupled with stereospecific class I diTPSs, miltiradiene synthase from *Salvia miltiorrhiza* (SmMS, ABV08817) [27] or kaurene synthase from *A. thaliana* (AtKS, AAC39443) [28], which respectively and specifically use CPP or *ent*-CPP. In this strain, the two class I diterpene synthases both produced ent-kaurene in assays of ApTPS4 and AtCPS coupled with AtKS, while miltiradiene was observed only in strain SmCPS1 coupled with SmMS. Thus, ApTPS4 was characterized as an *ent*-copalyl diphosphate synthase (Figure 3).

### 2.4. Identification of Recombinant Class I diTPS (TPS-e/f Subfamily)

To characterize the function of the putative class I diTPSs, class I diTPSs were expressed in BY-T20 coupled with either AtCPS or SmCPS1 (provided with *ent*-CPP or *nor*-CPP). The production was detected by GC-MS analysis (Figure 3B), only ApTPS5 could produce *ent*-kaurene in the same manner as that of AtKS, and miltiradiene was observed only in the extraction of SmCPS1 coupled with SmMS in BY-T20 strain fermentation products. No production was detected in the extraction of ApTPS6 and ApTPS7 coupled with AtCPS or SmCPS1 (Figure S2). The results demonstrated that ApTPS6 and ApTPS7 have no activity with either the *ent*-CPP or *nor*-CPP substrate. Thus, ApTPS5 was characterized as an *ent*-kaurene synthase.

### 2.5. Identification of Other Recombinant Class I TPSs (TPS-a/b/g Subfamily)

**Figure 4:**
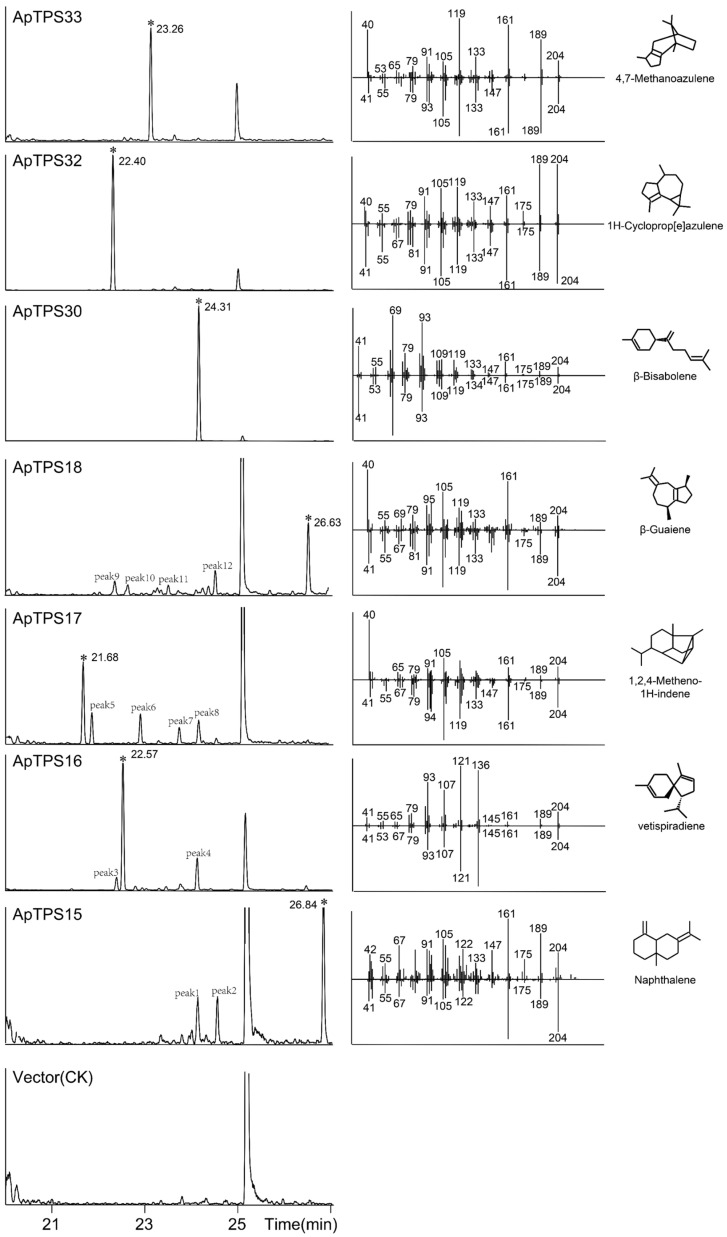
GC-MS analysis of the products from the extractions of *A. paniculata* TPS-a/b/g subfamily terpene synthase (in BY-T15) fermentation products (*: peak of the main products; peaks 1–13: the byproducts (Figure S4)).

To characterize the function of the putative TPS-a/b/g subfamilies of terpene synthases, their encoding genes were expressed in BY-15 or K197G [29] or coupled with ApCPS2 (providing *ent*-CPP) in BY-T20. GC-MS analysis of the extraction products (Figure 4) revealed no products in the K197G strain (GPP-producing) (Figure S3), indicating that no ApTPS candidates function as monoterpene synthases. Vetispiradiene, *β*-bisabolene, cyclopropazulene and 4,7-methanoazulene were detected in the fermentation extracts of ApTPS16, ApTPS30, ApTPS32 and ApTPS33 (BY-T15 strain), respectively. These results confirmed that ApTPS16, ApTPS30, ApTPS32 and ApTPS33 are vetispiradiene, *β*-bisabolene, cyclopropazulene and 4,7-methanoazulene synthases, respectively. Additionally, sesquiterpene products were detected in ApTPS15, ApTPS17 and ApTPS18 (BY-T15 strain). All products were identified by comparison to the NIST database and *m*/*z* data analysis (Figure 4 and Figure S4).

### 2.6. Key Residues and Site-Directed Mutagenesis of ApTPS

**Figure 5:**
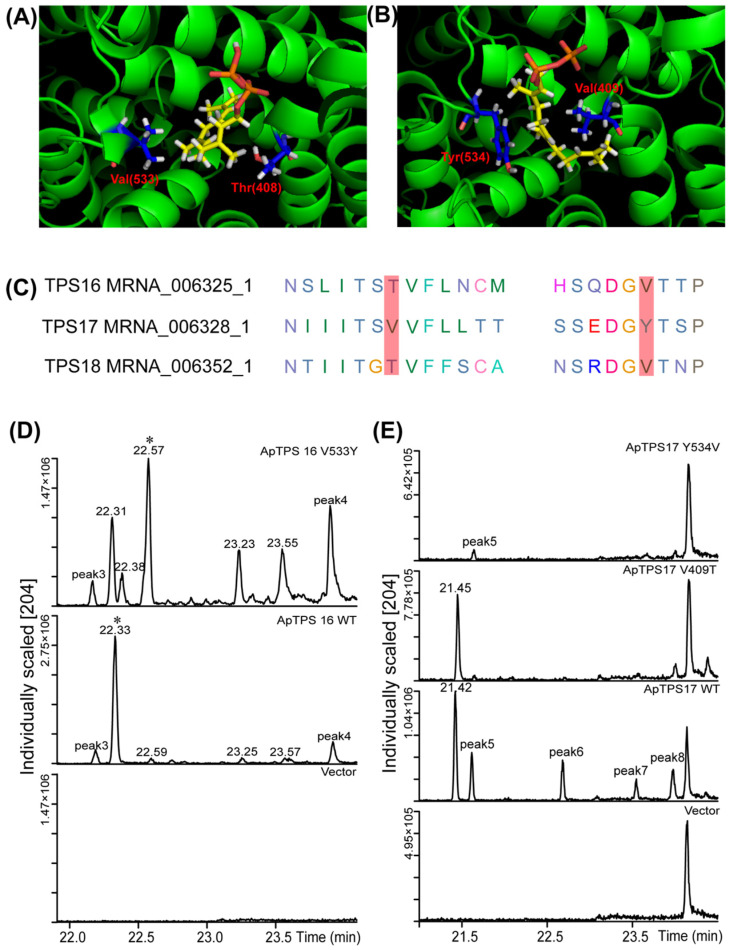
Key residues and site-directed mutants of ApTPS. (**A**) Docking of FPP in the active site of ApTPS16. (**B**) Docking of FPP in the active site of ApTPS17. (**C**) Conservation of select target residues in ApTPS16/17/18, as discussed in the text. (**D**) GC-MS analysis of extracted fermentation products of ApTP16 and their mutants (in BY-T15). (**E**) GC-MS analysis of extracted fermentation products of ApTP17 and their mutants (in BY-T15) (* peak of the main products; the ApTPS16 or ApTPS17 model is shown in green colour, the FPP ligand is shown as yellow sticks, and the target amino acids are shown as blue sticks).

By comparing genomic information, we found that ApTPS16, ApTPS17, ApTPS18 and ApTPS19 (ApTPS19 did not have enzymatic activity and was ignored in this study) were located on the same scaffold 2 and showed high homology according to phylogenetic analysis and protein homology (Figure 1, Tables S1 and S3). The crystal structures of 5-*epi*-aristolochene synthase from Nicotiana tabacum (tobacco) (PDB IDs: 4rnq, 4di5 and 5eat) were selected as suitable templates for ApTPS16, ApTPS17 and ApTPS18 in view of the high GMQE values (0.76, 0.75 and 0.75, respectively) of these enzymes and their high amino acid identities. Multiple sequence alignment analysis, homology modelling and molecular docking analysis of these three sesquiterpene synthases (Figure 5A,B) showed that ApTPS17 had a nonpolar amino acid (Val) at position 409, unlike ApTPS16 (and ApTPS18), which had a polar amino acid [30] at the same position (408). Consistent with these data, it was found that the ApTPS17 V409T mutant tended to make a single product compared with those made by WT ApTPS17, with the main product accounting for approximately 100% of the total products (Figure 5C), which suggests that V409 may be a key site for the diversification of ApTPS17 products. On the other hand, ApTPS16 (and ApTPS18) had a nonpolar amino acid (Val) at position 533, unlike ApTPS17, which had a polar amino acid (Tyr) at position 534; the ApTPS17 Y534V mutation caused the main product to disappear so that only one byproduct could be detected, compared with multiple products made by WT ApTPS17. Moreover, compared with the WT ApTPS16, the ApTPS16 V533Y mutation resulted in multiple main products.

## 3. Discussion

The phylogenetic analysis relative to the *A. thaliana* TPS gene family members (Figure 1) showed that 39 ApTPSs of *A. paniculata* were similar to those of *A. thaliana* (40 AtTPSs), while the number of diterpene synthase enzymes (members of the TPS-c and TPS-e/f subfamilies) in *A. paniculata* was much higher than that in *A. thaliana*. A total of ten diterpene synthases were screened from the *A. paniculata* genome, including four class II diterpene synthasess (CPSs) and six class I diterpene synthasess. By identifying the biological function of ApTPSs, we found that three of the class II TPSs (ApTPS1, ApTPS2 and ApTPS4) could convert GGPP to *ent*-CPP. *ent*-CPP is the direct precursor of andrographolide (the activity of ApTPS1 and ApTPS2 was analogous to that of ApCPS1 and ApCPS2) [19,20,25]. ApCPS4 is newly discovered here as an *ent*-copalyl diphosphate synthase that differs from ApCPS1, ApCPS2 and ApCPS3 [19,20,25]. Three of four class II diterpene synthases are *ent*-CPP synthases in the genome of *A. paniculata*; this provides a genetic explanation for the specific accumulation of *ent*-type labdane-related diterpenoids as the main chemical constituents in *A. paniculata*, which are the main specialized metabolites in *A. paniculata* (only the content of andrographolide was greater than 1%). ApTPS5 is only an *ent*-kaurene synthase whose sequence is the same as that of ApKSL2 [19,20,25], and its biological function is different from that of ApKSL2, which has multiple biological functions. Thus, some unknown factors may still exist causing the results to differ, and this needs further study.

The main chemical constituents of *A. paniculata* are labdane-type diterpenoids and flavonoids, while other components in the plant are rare. Sesquiterpenes and monoterpenes are important in plants but have not been reported in *A. paniculata*. We hypothesize that the content of other compounds may be present at low levels, as we failed to detect them in the original plant. Terpenoids are one of the largest groups of natural products in plants. However, only diterpene lactones of terpenoids have been reported in *A. paniculata* [31,32]. The present study provides the first comprehensive annotation of the enzymatic functions of members of the ApTPS gene family within the A. paniculata genome. By identifying the biological function of terpene synthases of *A. paniculata* to indicate the presence of other classes of terpenoids, we can study the chemical diversity in greater depth, which can also help us more effectively study the pharmacological activity of *A. paniculata*. In this study, we found seven multifunction or single-function sesquiterpene synthases (Figure 4 and Figure S1). These findings explain the diversity of the chemical composition by identifying the biological function of ApTPSs and provide evidence for the existence of *β*-bisabolene, cyclopropazulene, 4,7-methanoazulene and other sesquiterpenes, which could serve as a reference for future pharmacological research on *A. paniculata*. All monoterpene synthases in the type-b subfamily contain a conserved motif (RR(x)_8_W) downstream of their N-terminal transporters [33,34]. It is thought that the RR(x)_8_W motif is required for the conversion of GPP to cyclic monoterpenes [13,35,36], but only two of ten ApTPSs of the TPS-b subfamily contain the complete structure of RR(x)_8_W (Figure 2). The absence or substitution of amino acids within the conserved domain may cause the monoterpenoid synthase to lose its biofunction, which might be the reason that no accumulation of monoterpenoid compounds was seen in *A. paniculata*.

As a result of site-directed mutagenesis, ApTPS16 exhibited a nonpolar amino acid (Val) at position 533 compared to the wild-type (WT) ApTPS16. The mutation of this residue to a polar amino acid (Tyr) led to a diversification in product formation, resulting in the production of multiple products. In contrast, at position 534 of ApTPS17, a polar amino acid (Tyr) was found, and its mutation to a nonpolar amino acid (Val) resulted in the loss of the enzyme’s main biological function; only one byproduct could be identified in the ApTPS17 Y543V engineering yeast. The presence of the nonpolar amino acid 533Val in ApTPS16 and the polar amino acid 534Tyr in ApTPS17 are critical for maintaining each enzyme’s biological function. We propose that these specific sites may influence both substrate positioning and active site configuration, as well as affecting the stability of intermediates produced during terpene synthase catalysis.

## 4. Materials and Methods

### 4.1. Plant Materials

Seeds of *A. paniculata* were purchased from Zhangzhou, Fujian, China. The seeds were sterilized by antiseptic solution (2.0% sodium hypochlorite and 0.2% Tween 20) for approximately 10 min, washed five times with sterilized water, and sown on MS media (0.7% agar) for subsequent cultivation at 25 ± 2 °C under a 16/8 h (light/dark) photoperiod provided by an incandescent light bulb (3000 lux). After cultivating in MS media for two months, uniformly sized seedlings were selected and transferred into Hoagland solution (pH 5.8) to grow for two months under the same cultivation conditions [37].

### 4.2. Total RNA Isolation and cDNA Synthesis

Total RNA was extracted from the leaves, roots and stems of *A. paniculata* using a Quick RNA Isolation Kit (Thermo Scientific, CA, USA) according to the manufacturer’s instructions. A PrimeScript™ RT Reagent Kit with gDNA Eraser (Takara Bio., Beijing, China) was used to synthesize cDNA with 1 µg of total RNA according to the manufacturer’s protocol [37].

### 4.3. ApTPS Screening in A. Paniculata and Bioinformatic Analysis

To systematically identify TPS genes in *A. paniculata*, the genes were filtered according to their genomic annotation information. The nucleotide and deduced amino acid sequences were analyzed, and the sequences were compared using the BLASTP tool (version 1.4.0) on the NCBI website database (http://www.ncbi.nlm.nih.gov/, accessed on 14 April 2025). GSD tools (http://gsds.cbi.pku.edu.cn, accessed on 14 April 2025) was used to analyze the gene structures of the ApTPSs. MEME (version 5.5.7) (http://meme-suite.org/tools/meme, accessed on 14 April 2025) was then used to analyze the conserved motifs within the ApTPSs [38], with the default parameters. Multiple sequence alignment was implemented by ClustalW software (version 2.1, European Bioinformatics Institute), and MEGA 7.0 software was used to construct a phylogenetic tree using the NJ (neighbour-joining) method (bootstrap = 1000) [39]. The intron–exon structure and the conserved motifs of the TPSs were analyzed by DSDG (http://gsds.cbi.pku.edu.cn/, accessed on 14 April 2025).

### 4.4. Cloning of ApTPS Coding Sequences

Specific primers were designed based on the genomic sequence information to amplify the open reading frames (ORFs) of *ApTPSs*. All primers were designed using SnapGene software (version 7.0.2, snapgene.com) (all the primers used are listed in Table S2). A GeneJET Gel Extraction Kit (Thermo Scientific, CA, USA) was used to purify the PCR products, after which the products were cloned into a T-Vector (pEASY-Blunt Zero Simple Vector) (TransGen, Beijing, China), which was subsequently transformed into *Escherichia coli* DH5α (Covin Biosciences, Beijing, China) competent cells, and all operations were executed in strict compliance with the manufacturer’s guidelines. The reconstructed plasmids were purified, and their nucleotides were sequenced by Sangon Biotech Co., Ltd. (Shanghai, China).

### 4.5. Functional Characterization of Class II ApTPSs

The ORF regions of the *ApTPSs* were subcloned into a pESC-Leu expression vector via PCR amplification with a pEASY-Uni Seamless Cloning and Assembly Kit (TransGen, Beijing, China), and the amplicons were digested with NotI restriction enzymes (New England Biolab, Massachusetts, USA), and all operations were carried out according to the manufacturer’s protocol. The verified vectors were subsequently transformed into yeast strain BY-T20 (BY4742, Δ*Trp1*, *Trp1::HIS3-PPGK1-BTS1/ERG20-TADH1-PTDH3-SaGGPS-TTPI1-PTEF1-tHMG1-TCYC1*) [40]. The BY-T20 strain was transformed by integrating *BTS1/ERG20*, *SaGGPS* and *tHMG1* genes into the *Trp1* site of BY4742-Δ*TRP*. The BY-T20 cells harbouring the expression vector were selected on SD-Leu-His media (synthetic dextrose minimal media without leucine and histidine) (FunGenome, Beijing, China) (consisting of 20 g·L^−1^ glucose) and cultivated at 30 °C for 48 h in an incubator. The monoclonal plaques were identified in SD-Leu-His media at 30 °C for 48 h and were expanded at a rate of 1:100 into 100 mL of SD-Leu-His media (consisting of 20 g·L^−1^ glucose) under the same conditions for 48 h (OD_600_ = 2–3). The culture was centrifuged at 3000 g for 10 min to collect the cells, resuspended in yeast induction SD-Leu-His media (consisting of 20 g·L^−1^ galactose) and grown at 30 °C for 48 h in a shaking incubator [41]. Then, 1 mL of the yeast culture was pipetted from the cultured yeast suspension and mixed with an equal volume of *n*-hexane (Thermo Scientific, California, USA). The mixture was subjected to cell disruption using an ultrasonic disruptor. After disruption, the suspension was centrifuged at 13,000× *g* for 10 min, and the supernatant was collected. This process was repeated three times. The three aliquots of n-hexane were combined in an EP tube, dried under a stream of nitrogen (N_2_), and re-dissolved in 100 μL of *n*-hexane for GC-MS analysis [42]. The GC-MS analysis was performed using a Trace 1310 instrument (Thermo Scientific, California, USA) equipped with a TSQ 8000 mass detector and then separated with a TG-5 MS column (30 m × 0.25 mm I.D., DF = 0.25 μm (Thermo Scientific, California, USA). Helium was used as a carrier gas at a constant flow rate of 1.0 mL/min. The oven programme was as follows: 50 °C for 2 min, an increase of 20 °C min^−1^ to 300 °C, and then holding for 20 min. The ion trap temperature was 250 °C, and the scan range was 40 to 450 *m*/*z* [43]. The sample (1 μL) was injected in splitless mode.

### 4.6. Functional Characterization of Class I ApTPSs

The ORF regions of class I *ApTPSs* were subcloned with a pESC-Ura expression vector via PCR amplification with a pEASY-Uni Seamless Cloning and Assembly Kit, and the amplicons were digested with BamHI restriction enzymes. The vector constructs were verified by DNA sequencing and then transformed into yeast strain BY-T20 together with pESC-Leu::ApCPS2 or were transformed into yeast strain BY-T15 (BY4742, Δ*Trp1*, *PMET3-ERG9*, *rDNA::LEU2-PPGK1-tHMG1-TADH1-PTEF1-ERG20-TCYC1*) and K197G [29].

The BY-T20/BY-T15/K197G cells harbouring the expression vector were selected on SD-Leu-His-Ura/SD-Leu-Ura/SD-Trp-Ura media (FunGenome, Beijing, China) and subsequently grown at 30 °C for 48 h in an incubator. The culture and treatment conditions were consistent with those above, and only the media were changed (to SD-Leu-His-Ura/SD-Leu-Ura/SD-Trp-Ura), and was selected according to the screening tags of the plasmids within yeast.

The oven programme was as follows: 50 °C for 2 min, an increase of 20 °C min^−1^ to 300 °C, holding for 20 min or an increase to 60 °C for 1 min, an increase of 10 °C min^−1^ to 180 °C, an increase of 5 °C min^−1^ to 220 °C, an increase of 30 °C min^−1^ to 320 °C and holding for 3 min [44]. The sample (1 μL) was injected in splitless mode.

### 4.7. Homology Modelling and Molecular Docking

Homology modelling was used to construct the three-dimensional structure of ApTPSs within SWISS-MODEL Workspace (https://swissmodel.expasy.org/, accessed on 13 April 2025). After a search for templates in the PDB (https://www.rcsb.org/ accessed on 13 April 2025) based on alternative sequence alignments, templates were selected by their GMQW values, and several models were created based on the selected templates. The FPP substrate was refined using the elBow program in the PHENIX suite (version 1.14-3260). Modelling of ApTPS and FPP was then performed using molecular docking, as recently described [45]. The results of this docking run were shown with PyMOL (version 2.5.5).

## 5. Conclusions

To investigate the diversity of diterpenoids in *A. paniculata*, the genomic data were analyzed and candidate terpene synthase genes were identified based on functional annotation. Functional validation using a yeast expression system led to the identification of a novel *ent*-copalyl diphosphate synthase and six sesquiterpene synthases. Gene structure and conserved motif analyses revealed the likely causes of functional loss in most sesquiterpene and monoterpene synthases in *A. paniculata*, which may explain why diterpenoids are the dominant lineage-specific metabolites in this species. Molecular docking and site-directed mutagenesis further demonstrated that Val533 in ApTPS16 and Tyr534 in ApTPS17 are critical for their enzymatic activities. These findings provide new insights into the biosynthetic pathways of bioactive terpenoids in *A. paniculata*, offering valuable targets for metabolic engineering aimed at enhancing the production of active ingredients or generating novel terpenoids with potential pharmaceutical applications.

## Data Availability

The original contributions presented in this study are included in the article/supplementary material. Further inquiries can be directed to the corresponding authors.

## Supplementary Materials

The following supporting information can be downloaded at https://www.mdpi.com/article/10.3390/molecules30102208/s1, Figure S1: Map of the distribution of conserved motifs and the intron-exon structures of ApTPS; Figure S2: GC-MS analysis of the products from the extractions of ApTPS6 or ApTPS7 couple with AtCPS or SmCPS in BY-T20 (in BY-T20) fermentation products; Figure S3: GC-MS analysis of the products from the extractions of TPS-a/b/g subfamily terpene synthases (in K197G) fermentation products; Table S1: The information of ApTPSs and AtTPSs; Table S2: Primers used in this study; Table S3: Homology matrix of TPS-b subfamily ApTPS.

## Abbreviations

The following abbreviations are used in this manuscript:

TPS	terpene synthase
diTPS	diterpene synthase
GGPP	geranylgeranyl pyrophosphate
GPP	geranyl pyrophosphate
FPP	farnesyl pyrophosphate
CDS	the full-length coding sequences
CPS	copalyl diphosphate synthase
