# Supplementary material for: Characterization of Terpene Synthases Reveals the Diversity of Terpenoids in Andrographis paniculata
Source: Molecules. 2025 May 18;30(10):2208. doi: 10.3390/molecules30102208 (PMC12114178; doi:10.3390/molecules30102208)
Supplement: Supplementary file 1 [file molecules-30-02208-s001.zip › Supplimentary Information ApTPS.pdf]

Supplimentary Information

Characterization of terpene synthases reveals the diversity of terpenoids in *Andrographis paniculata*

Fig. S1 Map of the distribution of conserved motifs and the intron-exon structures of ApTPSs. **A** Map of the distribution of conserved motifs. **B** Map of the distribution of the intron-exon struc-tures.

Fig. S2 GC-MS analysis of the products from the extractions of ApTPS6 or ApTPS7 couple with AtCPS or SmCPS in BY-T20 (in BY-T20) fermentation products.

Fig. S3 GC-MS analysis of the products from the extractions of TPS-a/b/g subfamily terpene synthases (in K197G) fermentation products.

Table S1 The information of ApTPSs and AtTPSs

Table S2 Primers used in this study.

Table S3 Homology matrix of TPS-b subfamily ApTPS

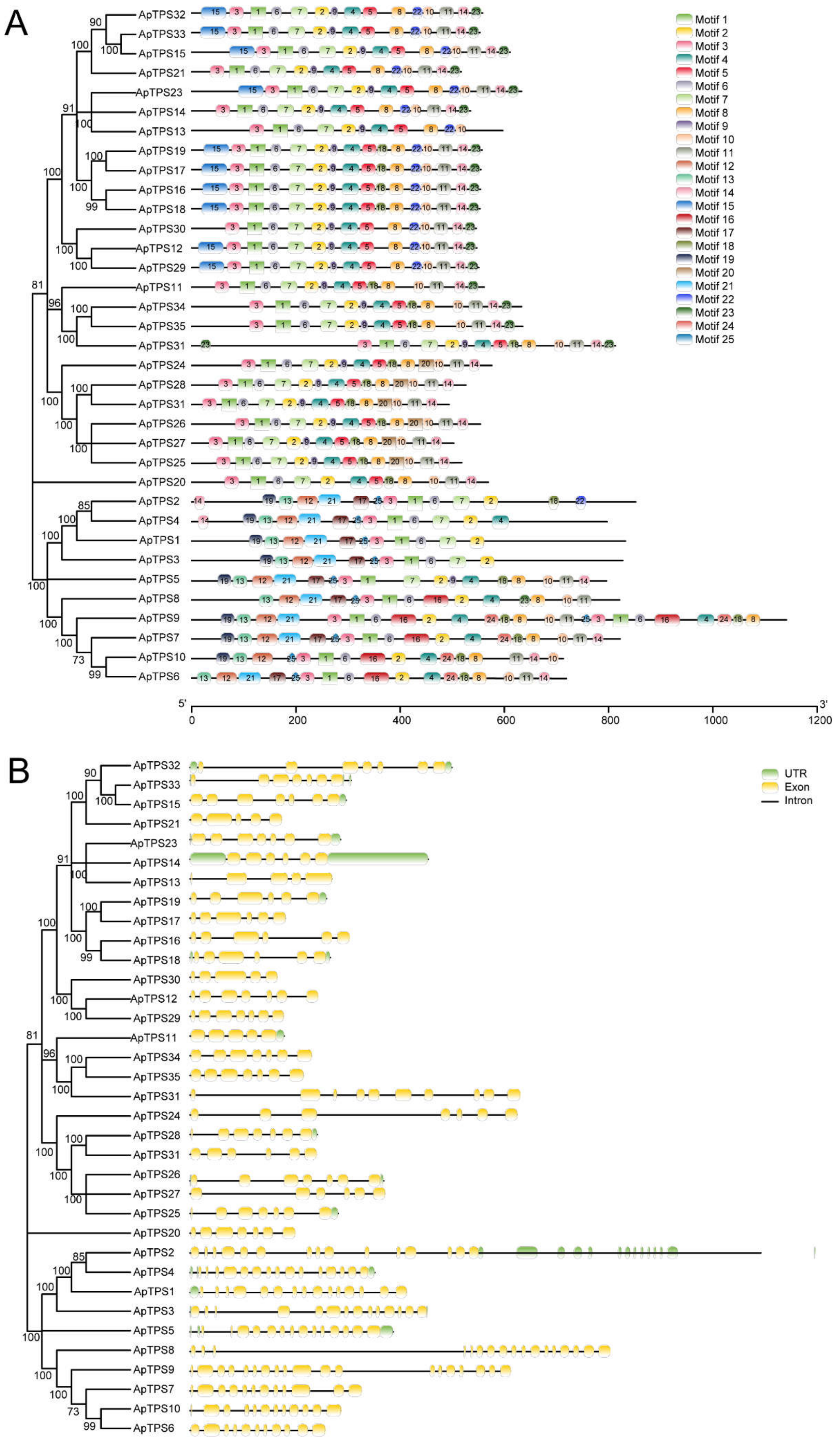
A
ApTPS32
ApTPS33
ApTPS15
ApTPS21
ApTPS23
ApTPS14
ApTPS13
ApTPS19
ApTPS17
ApTPS16
ApTPS18
ApTPS30
ApTPS12
ApTPS29
ApTPS11
ApTPS34
ApTPS35
ApTPS31
ApTPS24
ApTPS28
ApTPS31
ApTPS26
ApTPS27
ApTPS25
ApTPS20
ApTPS2
ApTPS4
ApTPS1
ApTPS3
ApTPS5
ApTPS8
ApTPS9
ApTPS7
ApTPS10
ApTPS6
Motif 1
Motif 2
Motif 3
Motif 4
Motif 5
Motif 6
Motif 7
Motif 8
Motif 9
Motif 10
Motif 11
Motif 12
Motif 13
Motif 14
Motif 15
Motif 16
Motif 17
Motif 18
Motif 19
Motif 20
Motif 21
Motif 22
Motif 23
Motif 24
Motif 25
5'
3'
0
200
400
600
800
1000
1200
B
ApTPS32
ApTPS33
ApTPS15
ApTPS21
ApTPS23
ApTPS14
ApTPS13
ApTPS19
ApTPS17
ApTPS16
ApTPS18
ApTPS30
ApTPS12
ApTPS29
ApTPS11
ApTPS34
ApTPS35
ApTPS31
ApTPS24
ApTPS28
ApTPS31
ApTPS26
ApTPS27
ApTPS25
ApTPS20
ApTPS2
ApTPS4
ApTPS1
ApTPS3
ApTPS5
ApTPS8
ApTPS9
ApTPS7
ApTPS10
ApTPS6
UTR
Exon
Intron
5'
0
2000
4000
6000
8000
10000
12000
14000

Figure S1. Map of the distribution of conserved motifs and the intron-exon structures of ApTPSs. **A** Map of the distribution of conserved motifs. **B** Map of the distribution of the intron-exon structures.

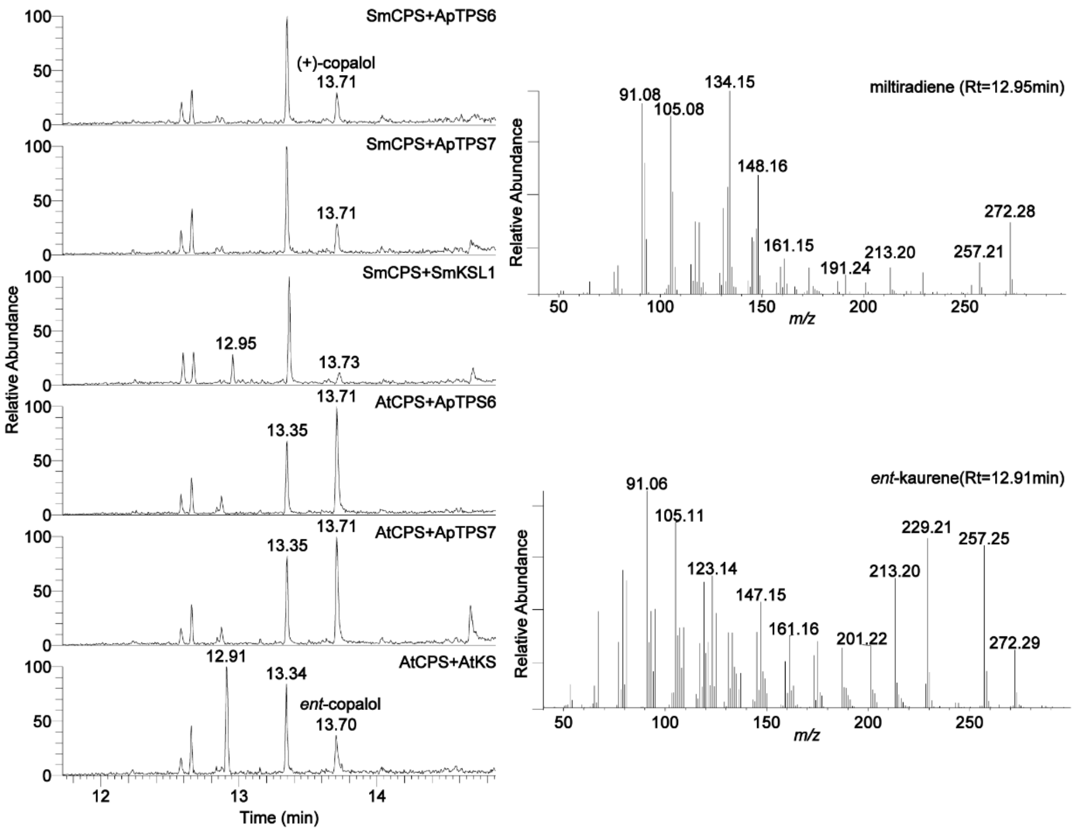

Figure S2. GC-MS analysis of the products from the extractions of ApTPS6 or ApTPS7 couple with AtCPS or SmCPS in BY-T20 (in BY-T20) fermentation products.

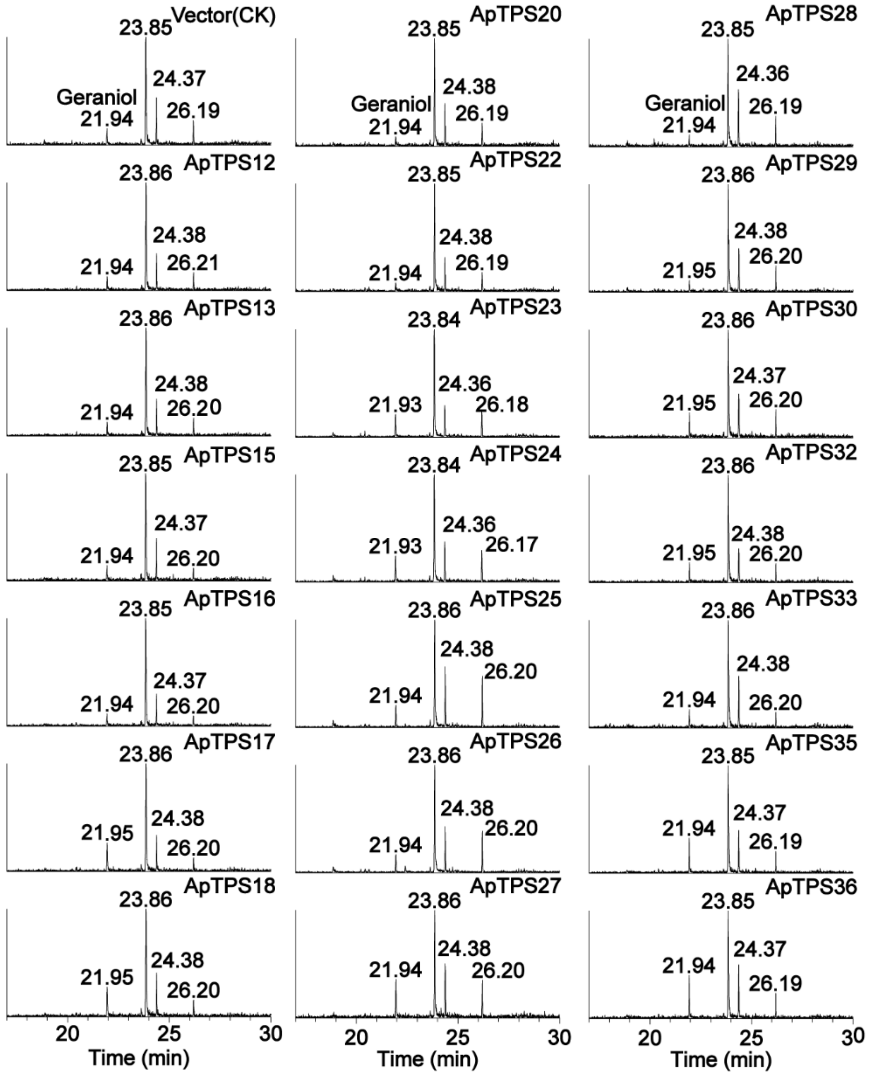

Figure S3. GC-MS analysis of the products from the extractions of TPS-a/b/g subfamily terpene synthases (in K197G) fermentation products.

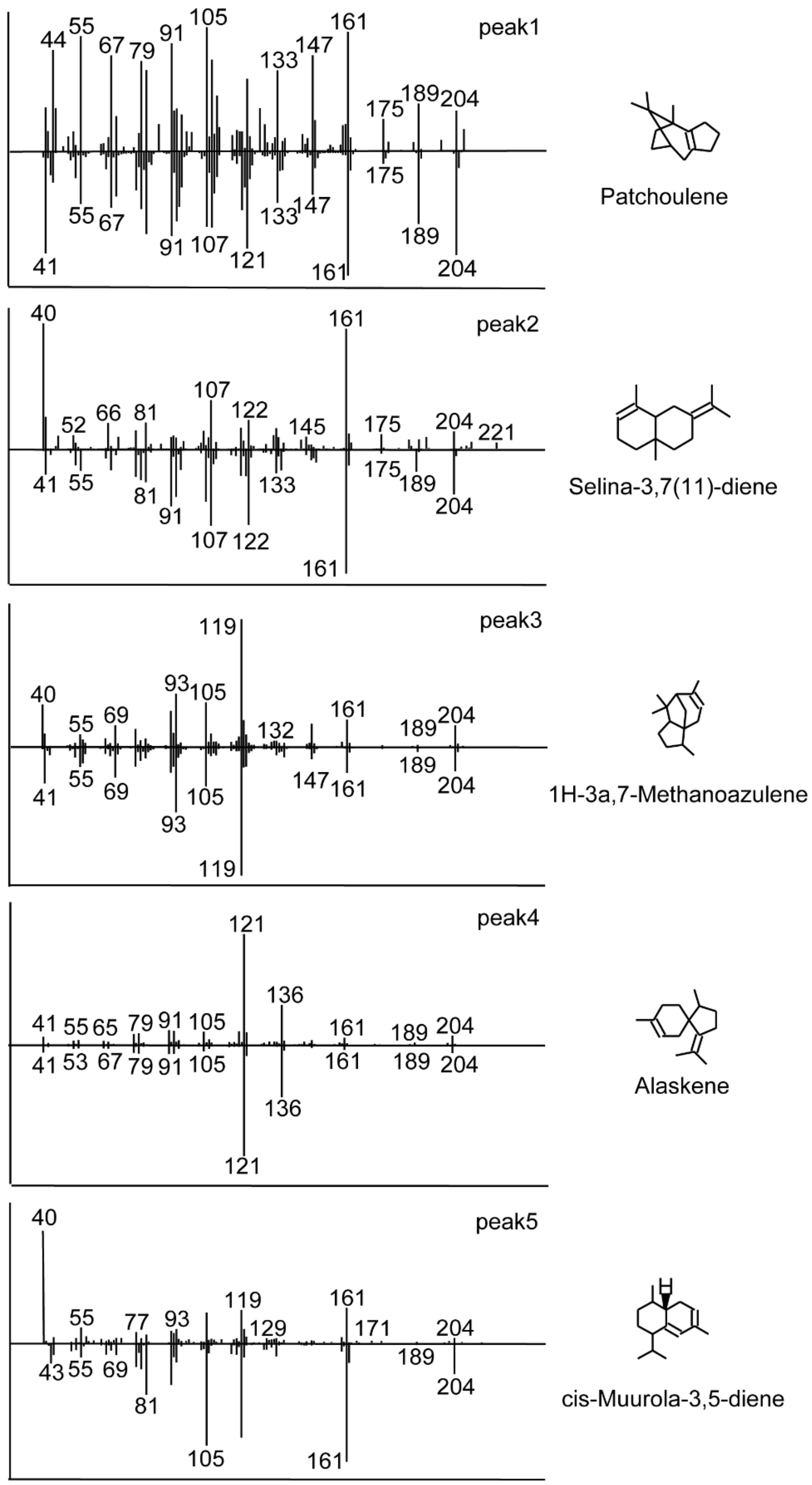
peak1
44 55 67 79 91 105 133 147 161 175 189 204
41 55 67 91 107 121 133 147 161 175 189 204
Patchoulene
peak2
40 52 66 81 107 122 145 161 175 204 221
41 55 81 91 107 122 133 161 175 189 204
Selina-3,7(11)-diene
peak3
40 55 69 93 105 119 132 161 189 204
41 55 69 93 105 119 147 161 189 204
1H-3a,7-Methanoazulene
peak4
41 55 65 79 91 105 121 136 161 189 204
41 53 67 79 91 105 121 136 161 189 204
Alaskene
peak5
40 55 77 93 119 129 161 171 204
43 55 69 81 105 161 189 204
cis-Muurola-3,5-diene

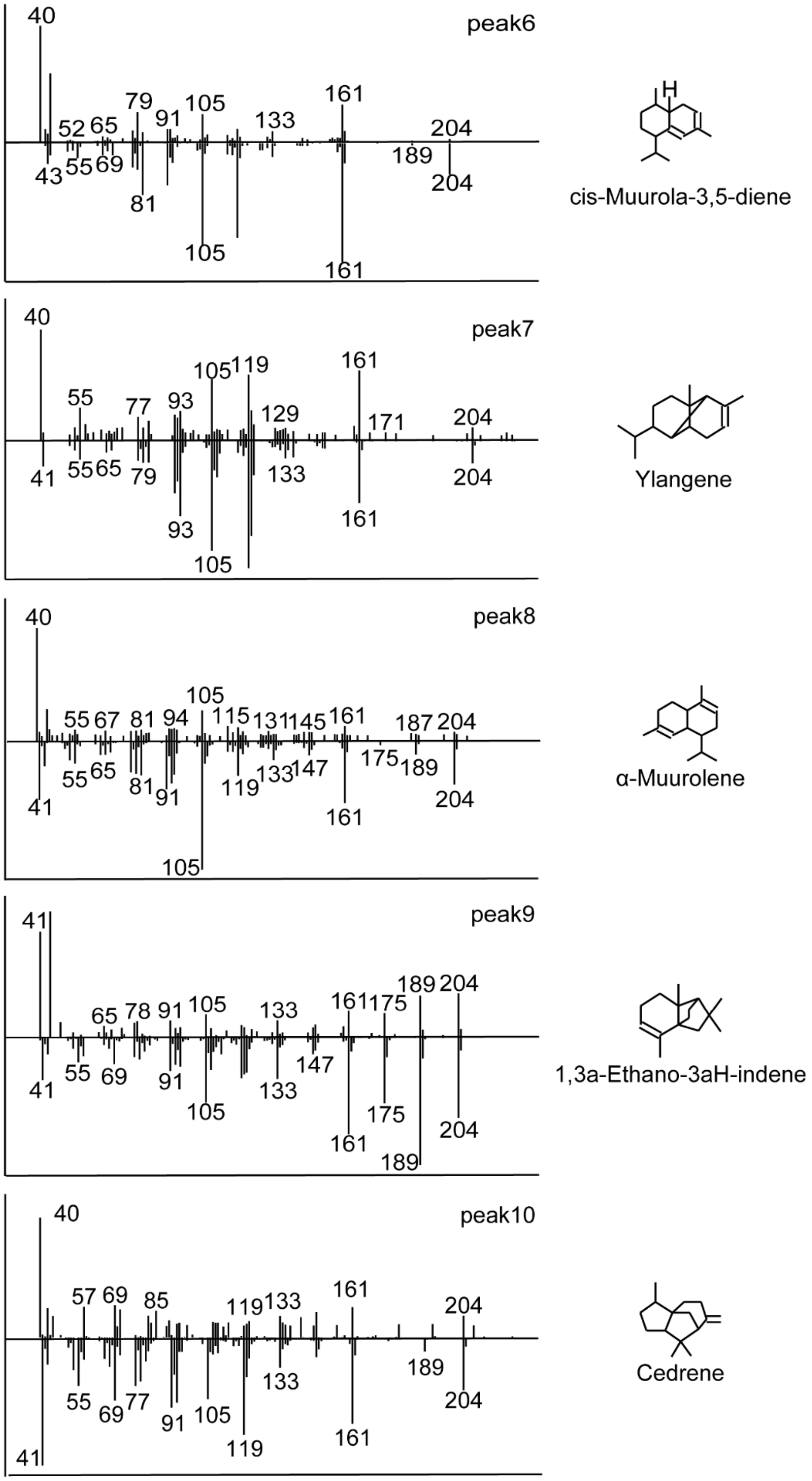
peak6
40 52 65 79 91 105 133 161 204
43 55 69 81 105 161 189 204
H
cis-Muurola-3,5-diene
peak7
40 55 77 93 105 119 129 161 171 204
41 55 65 79 93 105 133 161 204
Ylangene
peak8
40 55 67 81 94 105 115 131 145 161 187 204
41 55 65 81 91 105 119 133 147 161 175 189 204
α-Muurolene
peak9
41 65 78 91 105 133 161 175 189 204
41 55 69 91 105 133 147 161 175 189 204
1,3a-Ethano-3aH-indene
peak10
40 57 69 85 119 133 161 204
41 55 69 77 91 105 119 133 161 189 204
Cedrene

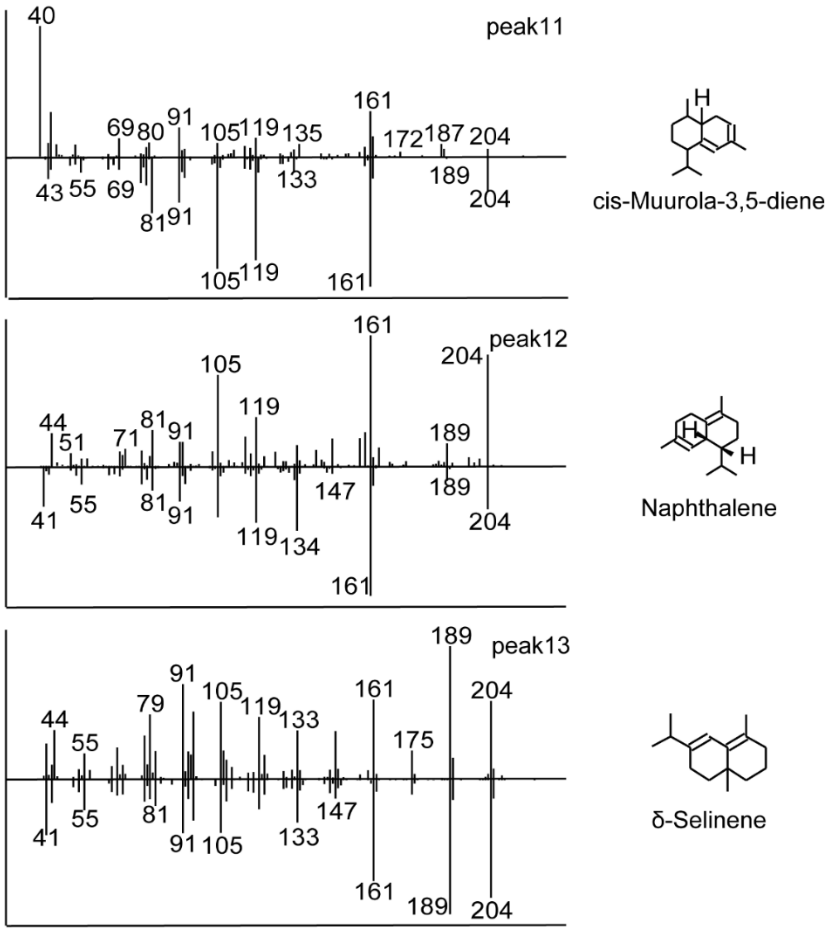

Figure S4. GC-MS analysis of the extraction by *A. paniculata* TPS-a/b/g subfamily terpene synthases (in BY-T15) fermentation products. (*m/z* of each peak)
